# iPSCs: A Minireview from Bench to Bed, including Organoids and the CRISPR System

**DOI:** 10.1155/2016/5934782

**Published:** 2016-01-06

**Authors:** Andrés Javier Orqueda, Carla Alejandra Giménez, Federico Pereyra-Bonnet

**Affiliations:** Basic Science and Experimental Medicine Institute, University Institute of the Italian Hospital, Juan D. Perón 4190, C1181ACH Buenos Aires, Argentina

## Abstract

When Dolly the sheep was born, the first probe into an adult mammalian genome traveling back in time and generating a whole new animal appeared. Ten years later, the reprogramming process became a defined method of producing induced pluripotent stem cells (iPSCs) through the overexpression of four transcription factors. iPSCs are capable of originating virtually all types of cells and tissues, including a whole new animal. The reprogramming strategies based on patient-derived cells should make the development of clinical applications of cell based therapy much more straightforward. Here, we analyze the current state, opportunities, and challenges of iPSCs from bench to bed, including organoids and the CRISPR system.

## 1. Introduction

Embryonic stem cells (ESCs) are pluripotent cells with a high self-renewal rate; they are derived from the inner cell mass of preimplantation embryos and can be differentiated into almost all cell types. The study of genes that are expressed in ESCs led to the identification of cell pluripotency-associated genes [1, 2]. In 2006, Professor Yamanaka's group isolated these candidate genes and introduced them in mouse fibroblasts cultured* in vitro* using viral vectors. Thus, the induced expression of these genes reprogrammed transfected cells, which became pluripotent and acquired ESC-associated morphology and gene expression pattern. These cells also gained the capacity to originate endodermal, mesodermal, and ectodermal tissues and differentiate into neural and cardiac cells [3]. For the first time, it was shown that it is possible to induce pluripotency* in vitro* in somatic adult cells with definite factors. These cells were first developed in mice and named induced pluripotent stem cells (iPSCs); subsequent research led to the development of human iPSCs, with clear impacts on therapeutic applications [4].

## 2. Genes and Mechanisms of Induced Pluripotency

Differentiated cells exhibit methylation and acetylation patterns that regulate gene expression and influence their potential development. Thus, in differentiated cells, most gene promoters are hypermethylated (typically associated with silenced chromatin), while, in stem cells, the opposite occurs, with most promoters being hypomethylated (typically associated with active chromatin) [5]. The differential pattern of methylation, acetylation, and ubiquitination of genes and histones is known as epigenetics. As discussed later, epigenetics involves transcription factors that are coded by genes used to obtain iPSCs, as well as proteins controlling the activation and repression of gene expression through the binding to the promoters of thousands of genes.

In the first study of iPSCs, 24 genes coding transcription factors related to ESCs were introduced into mouse fibroblasts [3]. The expression of those factors led to cell reprogramming, reaching a pluripotency state similar to that of ESCs. Surprisingly, the results show that the induced expression of only four factors was necessary for reprogramming pluripotency: OCT4, SOX2, c-MYC, and KLF4, commonly named Yamanaka's factors. The mechanism of action of these factors in iPSCs has been only partially elucidated.

Reprogramming is governed by slightly different mechanisms according to cell type. OCT4, SOX2, and NANOG constitute the core transcriptional regulatory circuitry that allows pluripotency and self-renewal in human ESCs [2]. However, the addition of the* NANOG* gene is dispensable for the generation of human iPSCs [4]. OCT4 and SOX2 usually have actions on the same human ESC promoters, probably cooperatively. Thus, the OCT4/SOX2 complex is believed to act as a key regulator that controls the expression of developmental genes. In addition, several NANOG binding sites are found in the same sequences, such as OCT4/SOX2 complex binding sites, indicating the likely complexity of their interrelationship in gene regulation. Thus, OCT4 directly binds 623 promoters related to several protein-coding genes. SOX2 and NANOG have been found in association with 1271 and 1687 promoters, respectively. Of that number, 353 are regulated by OCT4, SOX2, and NANOG altogether [2]. Surprisingly, the autoregulation of* OCT4*,* SOX2,* and* NANOG* by self-binding to their own promoters has also been reported. Together, these observations suggest that OCT4, SOX2, and NANOG promote pluripotency, cell self-renewal, and suppression of cell differentiation programs.

The KLF4 and c-MYC transcription factors are also used to obtain iPSCs. The high expression of* Klf4* and* c*-*Myc* has been identified in tumors; thus, these genes are related to cell proliferation and self-renewal. In mammalian genomes, there are more than 25,000 putative c-MYC binding sites, highlighting its role [6]. Meanwhile, KLF4 suppresses the* p53* gene, and because the p53 protein inhibits* NANOG* gene during ESC differentiation, KLF4 could function by activating* NANOG* through* p53* inhibition. Another factor that is commonly used to obtain iPSCs is LIN28, which is related to the negative regulation of the processing of microRNAs that act on the cell differentiation of ESCs [2]. This effect on microRNA repression has also been proposed for c-MYC.

Previous studies cited here have demonstrated the mode of action of transcription factors in gene regulation but do not explain how these factors modify chromatin remodeling. However, different reports have gradually provided significant data to begin uncovering these mechanisms. For example, OCT4 binding to specific targets has been associated with the ability to recruit p300 histone acetyltransferase, showing the interrelationship between transcription factors and modifying histones proteins [5]. In addition, c-MYC has been shown to be associated with the p300 complex [7] and is believed to act on the global acetylation of histones, allowing, for example, OCT4 and SOX2 to bind to their targets [3]. It is well known that several levels in the control of gene expression exist; among these levels, the initiation of transcription involves chromatin architecture and the access of transcription factors to the target site. Thus, DNA hypermethylation catalyzed by DNA methyltransferases in promoters usually silences chromatin and inhibits transcription, while the hypomethylated status commonly associates with actively expressing chromatin. On the other hand, DNA-associated histones may be acetylated and/or methylated, driving different patterns of expression that highly vary according to the residues, histones involved, and multiplicity of amino acids modified. For example, lysine 4 from histone 3 can be trimethylated, generally leading to an active status of chromatin, while lysine 27 from histone 3 can be trimethylated, leading to inactivating chromatin. The acetylation of histones is catalyzed by histone acetyltransferases (HATs), which activate chromatin; in contrast, deacetylation is mediated by histone deacetylases (HDACs), which silence the chromatin.

Altogether, these results allow researchers to develop new strategies to artificially induce cell pluripotency reprogramming. Some recent reports indicate that only 3 factors (OCT4/SOX2/NANOG) are needed to derive human iPSCs from somatic adults cells [8]. Additionally, only 2 factors are needed when the histone deacetylase inhibitor, valproic acid, is added [9]. The effects of c-MYC can be partially compensated for by valproic acid, demonstrating that c-MYC modifies histone acetylation. Valproic acid can replace KLF4 functions in human cell reprogramming, demonstrating that c-MYC and KLF4 may affect similar mechanisms of control. Interestingly, recent studies suggest that OCT4, which was previously believed to be irreplaceable, can be substituted in some cell types by chemically inhibiting G9a histone methyltransferase [5]. These results drive the development of a plethora of next-generation pluripotency inducers, such as pharmacological drugs (discussed below). Notably, unraveling pluripotency reprogramming mechanisms will provide data to better understand cell regulation and related conditions, such as cancer and cell ageing.

## 3. Methods of Producing iPSCs

iPSCs were first obtained through the transfection of mice and human fibroblasts with viral vectors [3, 4]. This procedure is based on the capacity of retroviral and adenoviral vectors to efficiently introduce genes inside the nucleus. Viral vectors lack pathogenic genes from viruses and only possess information for the packaging and integration of the transgene in the host genome. In addition, these vectors contain reprogramming genes (usually* OCT4*,* SOX2*,* KLF4,* and* c-MYC*) with strong downstream promoters leading to high levels of expression.

There are advantages and disadvantages when these vectors are used: on the one hand, they are characterized by a high efficiency of integration (60 to 80% in the case of retroviruses) and can be used in cells with low mitotic rate or even in nondividing cells (lentiviruses). On the other hand, there is a strong requirement for biosafety protocols when handling viral vectors, and they have low potential in clinical trials.

With the aim of avoiding the integration of foreign genes into the human genomes because of known ethical issues, assays with iPSCs have begun to use new strategies based on the expression of pluripotency genes without being integrated. Thus, the use of circular plasmids and messenger RNA (mRNA) emerged; circular plasmids are 10 kbp molecules that are closed by covalent bonds. These molecules are easily handled and can be driven inside cells through liposomes or cell pores that are generated by electroporation. One study showed that iPSCs can be obtained from human fibroblasts cultured* in vitro* using a circular plasmid coding OCT4, SOX2, LIN28, NANOG, and Green Fluorescent Protein (GFP) reporter genes [10]. Two weeks after transfection, green cells started acquiring ESC-like features and stopped expressing GFP. Later, PCR genetic analyses revealed that once iPSC lines were established, there were no more traces of the plasmid containing foreign genes, suggesting the following: (i) plasmids were not integrated in the human genome and were lost during successive mitotic divisions and (ii) the expression of foreign transcription factors was only necessary during the first steps of cell differentiation, leading then to the expression of endogenous transcription factors.

More recently, human fibroblasts were reprogrammed with mRNA coding the main 4 factors [11]. The authors demonstrated the inefficiency of this methodology because of the need for 5 cycles of reprogramming with mRNA encoding the 4 transcription factors to achieve iPSCs. However, this strategy presents a major advantage in a context of approaching the bench to the bed: it does not require DNA sequences and does not modify genetic host cells. Consequently, it is expected that these alternative technologies will gain high relevance for future clinical applications.

Some years ago, the exogenous expression of pluripotency-associated factors (at least OCT4) was believed to be indispensable for establishing pluripotency [12]. However, a chemical reprogramming strategy emerged with great potential use in generating functional and desirable cell types, excluding genetic manipulation, which limits clinical applications [13]. This type of reprogramming is based on cell-permeable and nonimmunogenic small molecules, which are often easily synthesized and more cost-effective; interestingly, their effects rely on reversible inhibition or the activation of specific protein functions. In this context, the identification of small molecules driving the reprogramming of mouse embryonic fibroblasts has recently been reported [14]. Thus, the glycogen synthase kinase 3 inhibitor CHIR, the transforming growth factor-*β* inhibitor 616452, the cAMP agonist Forskolin, and the S-adenosylhomocysteine hydrolase inhibitor DZNep were identified in a small-molecule library and demonstrated induced expression levels of most pluripotency marker genes and growth, with a doubling time similar to that of ESCs. Importantly, the DNA methylation state and histone modifications at* OCT4* and* NANOG* promoters in chemically iPSCs were similar to those in ESCs; in addition, differentiation into tissues of all three germ layers was observed when reprogrammed cells were injected into immunodeficient mice, showing fully reprogramming ability.

In this line of evidence, other pathways involved in reprogramming include MEK and transforming growth factor-*β*. One study showed that reprogramming can also be achieved with chemical inhibitors of the MEK and transforming growth factor-*β* pathways (PD0325901 and SB431542, resp.) [15]. In this study, the specificity of different primary cell cultures to both inhibitors was reported; iPSCs were generated only from the head-derived primary culture of mouse embryonic cells, while primary cell cultures that were derived from the liver, side-body skin, and tail-tip of the embryos showed no reprogramming.

Unfortunately, until now, small-molecule strategies have shown little efficiency in reprogramming. For example, a CHIR/616452/Forskolin/DZNep combination generated iPSCs from mouse somatic cells at a frequency of up to 0.2% [14]. In addition, an estimated efficiency of the reprogramming of 4 iPS-like colonies per 1.7 × 10^6^ starting cells has been reported, of which 40% of clones were alkaline-phosphatase-positive, a characteristic feature of ESCs [15]. As a consequence, great efforts have been made to obtain a higher efficiency in reprogramming strategies. One study recently showed an almost 100% efficiency in reprogramming somatic cells. By means of genetic depletion of the core member of the nucleosome remodeling and deacetylation complex* MBD3*,* OCT4/SOX2/KLF4/c-MYC* transgene delivery, inhibition of ERK1/2 and GSK3-*β*, and stimulation with leukemia inhibitory factor (LIF), pluripotency was achieved in mouse embryonic fibroblasts, which showed similar genome-wide chromatin mapping for H3K27me3, H3K4me3, and H3K27ac histone markers, genome-wide DNA methylation mapping, and expression of key endogenous pluripotency markers to that of ESCs [16].

## 4. Identification and Characterization of iPSCs

At first glance, iPSCs are very similar to ESCs: they form flat colonies with regular borders, divide at similar rates, self-renew (allowing a great number of passages* in vitro*), and exhibit long nucleoli and a limited cytoplasm. Although morphological evidence is mandatory, characterization requires other techniques [17], such as those based on the analysis of immunofluorescence, the expression pattern, and the capacity for the generation of different tissue types (pluripotency).

One of the first tests that were developed to determine cell pluripotency is the alkaline phosphatase activity assay because this enzyme is active in ESCs and iPSCs. The telomerase-coding gene* hTERT* is another gene that is typically expressed in these types of cells; this enzyme lengthens chromosome ends (telomeres), assuring indefinite cell division. On the other hand, immunocytochemical techniques are utilized to fluorescently label membrane and intracellular proteins, as well as transcription factors such as OCT4, SOX2, and NANOG, which are associated with ESCs. For these techniques, cells must be fixed, permeabilized, and incubated with specific antibodies against OCT4, SOX2, NANOG, and so forth; subsequent observation of samples through fluorescence microscopy allows for the detection of these proteins. These genes can also be assayed by western blotting or real-time PCR following retrotranscription.

Importantly, obtained iPSCs must be demonstrated to originate the 3 germ layers [1, 3, 17]. One method of choice is suspension culture forming embryoid bodies [18, 19]. Embryoid bodies are round cell clusters that are obtained when iPSCs are cultured in suspension, transferred to gelatin-coated plates, and cultured afterwards until the appearance of adherent cells of endo-, meso-, and ectodermal origin. The formation of teratomas is another assay that is frequently used to study the pluripotency of iPSCs [20]. Teratomas are tumors containing a variety of cell types, which can originate a number of tissues, for example, cartilage, skin, hair, and even nails. These tumors are usually generated by the subcutaneous injection of iPSCs in immunosuppressed mice.

Chimera formation is another test for the pluripotency of iPSCs that can only be assayed in animals and nonhuman primates. Chimeras are made by the addition of iPSCs to embryos that were previously obtained by fertilization; after chimeric embryos are transferred to the receptor females in which they will develop, the contribution of iPSCs to the different tissues of the newborn animals is analyzed. However, iPSCs generate live chimeras in mice only with great difficulties [3].

Finally, despite global gene expression and methylation and acetylation markers to identify iPSCs, it is now well established that these patterns may deeply differ from those of ESCs, highlighting the differences between both types of cells [4, 21–24].

## 5. Miniorganoids

Recent breakthroughs in 3-dimensional (3D) organoid cultures of many organ systems have led to new* in vitro* physiologically complex models. Perhaps in this new scenario, the engineering of human organs will take greater advantage of iPSCs, furthering the study of human development and disease transplantation. The coculturing of more than one iPSC-derived cell type to make complex autologous* bona fide* organs for transplantation medicine may be achievable in the near future. Meanwhile, iPSCs have demonstrated the ability to generate organoids that are capable of making functional 3D structures for* in vitro* disease modeling and drug screening.

### 5.1. Miniature Stomach

Gastric diseases, including peptic ulcer disease and gastric cancer, affect 10% of the world's population and are largely due to chronic* Helicobacter pylori* infection. In a recent report, the authors showed that the temporal manipulation of several signaling pathways (FGF, WNT, BMP, retinoic acid, and EGF) and 3D growth are sufficient to generate human gastric organoids (3D human gastric tissue* in vitro*) from human iPSCs [25]. The organoids formed a primitive gastric gland, surface cells, antral mucous cells, and a diverse amount of gastric endocrine cells. Interestingly, these organoids were successfully used for modeling* H. pylori* infection.

### 5.2. Growing a Gut

A robust and efficient process to direct the differentiation of human iPSCs into 3D gut organoids has also been created [26]. The resulting intestinal 3D-cultured tissue presented a cellular composition similar to that of fetal intestine, expressed intestinal stem cell markers, and presented absorptive and secretory functions. The epithelium contained functional enterocytes, as well as goblet, Paneth, and enteroendocrine cells. In addition, the interaction between the human iPSCs-derived intestinal organoids and* Salmonella enterica* populations has been explored [27], demonstrating that iPSC-derived organoids are promising models of intestinal epithelium.

### 5.3. Minilivers

Recently, the generation of vascularized and functional human liver from human iPSCs through the transplantation of liver buds that were created* in vitro* was reported [28]. Interestingly, specified hepatic cells self-organized into 3D iPSC-derived organoids. Immunostaining and gene expression analyses revealed similarities between iPSC-derived organoids and* in vivo* liver buds. In addition, human vasculatures in iPSC-derived organoid transplants became functional by connecting to the host vessels. The highly metabolic iPSC-derived tissue performed liver-specific functions, such as protein production and human-specific drug metabolism, without recipient liver replacement; in addition, the mesenteric transplantation of these organoids rescued a drug-induced lethal liver failure model, demonstrating for the first time the generation of a functional human organ from iPSCs.

### 5.4. Little Lungs

A recent report has demonstrated the step-wise differentiation of human iPSCs into lung organoids. After the manipulation of the signaling pathways that are involved in development, iPSCs generated lung organoids consisting of organized compartments and showing structural features similar to those of the native lungs. In addition, the lung organoids possessed an upper airway-like epithelium with basal and immature ciliated cells that were surrounded by smooth muscle and myofibroblasts, as well as an alveolar-like domain. Based on global transcriptional profiles, the authors demonstrated that lung organoids are remarkably similar to human fetal lungs [29].

### 5.5. Baby Brains

Cerebral organoids have been made by the 3D culturing of neuroectoderm derived from human iPSCs [30, 31]. This method can give rise to a developing cerebral cortex, ventral telencephalon, choroid plexus, and retinal identities, among others, within 1-2 months. Furthermore, because organoids can be maintained for more than 1 year in long-term culture, they also have the potential to model later events, such as neuronal maturation and survival.

### 5.6. Building Hearts

The ability to create whole functional hearts by means of tissue bioengineering has proven elusive. The closest result to this bioengineering task has been the engineering of heart constructs by repopulating decellularized mice hearts with human iPSC-derived multipotential cardiovascular progenitor cells [32]. The authors reported that the seeded cells migrated, proliferated, and differentiated* in situ* into cardiomyocytes, smooth muscle, and endothelium. After 20 days of perfusion, the engineered heart tissues exhibited spontaneous contractions, generated mechanical force, and were responsive to drugs. These novel results will benefit the study of early heart formation and contribute to the search of applications in preclinical testing.

### 5.7. Tiny Eyes

Under defined culture conditions, iPSCs have been used to generate optic vesicle-like structures that generate retinal cell types [33] that are suitable for* in vitro* studies and disease modeling [34]. Thus, iPSCs were differentiated into 3D optical vesicles and later started expressing markers of intercellular communication. These 3D optical vesicles contained multiple neuroretinal cell types and spontaneously formed primitive laminae, reminiscent of the developing retina, demonstrating the capacity of iPSCs to self-assemble into rudimentary neuroretinal structures. As a proof-of-principle, the authors examined the role of a key transcription factor, visual system homeobox 2 (VSX2), using iPSC-derived optic vesicle-like structures that were obtained from a patient with microphthalmia caused by an R200Q mutation in the VSX2 homeodomain region. iPSC-derived vesicles have emerged as a versatile model system to study retinal development at stages that were not previously accessible in humans. Finally, the use of retinal pigmented epithelial cells that were derived from iPSCs to treat eye disease is currently being evaluated in clinical trials (see below).

Table 1 summarizes the miniorganoids that have been obtained up to now and the associated modeling of diseases.

In summary, iPSC technology has driven the creation of 3D structures that resemble tissues from the eye, gut, liver, lung, stomach, and brain, among others. These engineered organoids mimic some structures and functions of real organs, increase knowledge of human development, provide novel tools for drug-screening platforms, and serve as disease models that would eventually replace animal models. Consequently, it is possible to envision the use of personalized iPSC-derived organoids in clinical trials. However, the generation of complex 3D organs* in vitro* remains a major challenge for translational studies.

## 6. Impact on Cell Therapy

The discovery of the reprogramming and differentiation of human adult cells has generated great expectations because of potential therapeutic applications. iPSCs have a major advantage in regenerative therapies because they can be obtained from the same patient, avoiding immune rejection when transplanted. Additionally, culturing iPSCs under restrictive conditions can lead to differentiation to specific cell types (Table 2).

The first attempts came from animal model studies, providing promising results. There have been several assays reporting the therapeutic use of iPSCs in preclinical trials assayed in animals, mainly mice. This technology has led to the reversal of hyperglycemia in diabetic mice [35]. Then, assays in animals presented evidence supporting the potential application of iPSCs in cell therapy. This and other recently achieved goals are shown in Table 3.

Studies in human samples have also produced interesting results. For example, the differentiation of iPSCs into insulin-producing pancreatic cells has been reported [35, 41]. This* in vitro* differentiation requires culture media with proper growth factors and chemicals, such as activin-A [42] and sodium butyrate [43]. After 3 weeks under these conditions, cells start clustering in pancreatic-like islets and secreting insulin after glucose stimulation. The results of these studies demonstrate that pancreatic cells can be obtained from the skin of diabetic patients.

Another promising example is the formation of human motor neurons from iPSCs [48]; in this report, the authors showed that motor neurons can be obtained when iPSCs are cultured under retinoic acid treatment and challenged with Sonic Hedgehog pathway agonists and neurotrophic factors. As a result, iPSC-derived motor neurons express typical molecular markers and are electrically active, similar to those obtained from human ESCs.

The transplantation of iPSCs for cell therapy in humans should overcome the following obstacles: (i) avoidance of the integration of foreign DNA in the human genome (alternative methodologies have been described above, e.g., using plasmids, mRNA, or small molecules); (ii) avoidance of the risky use of oncogenes during the induction of pluripotency (e.g.,* Klf4* and* c-Myc*); and (iii) replacement of animal-origin products in the media to avoid possible zoonoses.

*First Pilot Study*. Age-related macular degeneration is one of the most common causes of visual impairment in the elderly, and protocols for the formation of human retinal pigment from iPSCs have been previously developed, including the use of scaffolds [49, 50]. Similarly, nonhuman retinal pigment has been obtained from iPSCs [51]. Importantly, one study showed that iPSC-derived retinal pigment epithelium resembles native retinal pigment epithelium according to the similar expression of typical retinal pigment epithelium markers, the formation of tight junctions with the polarized secretion of growth factors, phagocytic ability, and gene expression patterns [50]. In addition, in this report, these authors demonstrated that transplanted autologous nonhuman primate iPSC-derived retinal pigment epithelium cell sheets show no immune rejection or tumor formation. Along the same line of evidence, it has been shown in an immunodeficient mice model that iPSC-derived retinal pigment epithelium has negligible tumorigenic potential [52].

The treatment of age-related macular degeneration has recently witnessed great advances in a first pilot study. The first attempt to study the safety and feasibility of transplanted retinal pigment epithelium cell sheets in patients with exudative age-related macular degeneration took place in Japan. Autologous iPSC-derived retinal pigment epithelium cell sheets were transplanted into an elderly age-related macular degeneration volunteer. iPSCs and subsequent retinal pigment epithelium cell sheets were produced and validated at a certified clinical grade before transplantation, leading to the posterior analysis of the functional integration of retinal sheets and potential adverse reactions. Unfortunately, a later analysis revealed 6 mutations in the patient's cells, one of them in an oncogene, although associated with low risk tumorigenesis. Tt is believed that these mutations occurred when iPSCs were manipulated, during their isolation or differentiation [53].

## 7. Application of iPSCs and CRISPR/Cas9 Technology

Although the application of iPSC technology in humans has increased in frequency as a promising regenerative therapy, a new field based on the* in vitro* use of human iPSCs in diseases modeling has emerged due to the capacity to test new chemicals or patient-specific treatments (as discussed above). The possibility of using human cells that are patient specific has high potential. iPSCs proliferate indefinitely* in vitro* and can be differentiated to almost any cell type of the human body, such as cardiomyocytes, nerve cells, or insulin-producing pancreatic cells, providing excellent foundations as human models for testing drug efficiency or toxicity [54].

In addition to patient-specific treatments, there is great interest in developing human iPSC lines with a particular genotype characteristic of certain diseases to understand different pathogeneses. The use of cells from patients who carry the altered genetic background can be considered an adequate option to establish a desired iPSC line. However, it cannot be ruled out that other differences between healthy control and disease genotypes may drive phenotypic differences [55]. Fortunately, new technologies based on iPSCs and CRISPR/Cas9 gene editing have emerged as important tools due to their capacities of generating a cell phenotype with a specific gene failure to be studied* in vitro* using an isogenic cell line as a control [56].

CRISPR/Cas9 is a defense system in which bacteria and archaea degrade viral DNA by means of an RNA probe that is complementary to a target sequence and a nuclease protein (Cas9) [57]. The use of CRISPR/Cas9-derived biotechnology became a practical RNA-guided platform for targeting and cutting any specific DNA loci by simply specifying a 20 nt targeting sequence within its RNA probe [58–60]. The adaptation of this system for use in eukaryotic cells has resulted in a milestone in the history of genetic engineering because CRISPR/Cas9 technology is cheaper and easier to use than its predecessor techniques involving TALENs and Zinc fingers [61].

In terms of the special capabilities of gene editing for disease modeling, CRISPR/Cas9 technology allows the knock-out of one or more genes at once [62], as well as the knock-in of specific alleles in iPSCs that are associated with different diseases, utilizing single-stranded DNA oligonucleotides as templates for homology-based repair [63]. Previously, gene-editing approaches employed randomly integrating viruses with concomitant issues of insertion mutagenesis, inaccurate gene dosage, and gene silencing, which are inconvenient for clinical application [64]. It must be noted that CRISPR/Cas9 editing occurs at a specific DNA locus of interest, avoiding viral vectors-associated random insertion. In terms of its application in cellular models, the success of the combination of iPSCs and CRISPR/Cas9 technologies in* in vitro* modeling lays in (i) the ability of CRISPR/Cas9 to rapidly and precisely edit genes; (ii) the capacity of iPSCs to proliferate indefinitely, allowing a highly efficient selection of clones carrying the gene modification; and (iii) the capacity of this cell type to reprogram into the desired cell phenotype after DNA editing. The progress has been so high that a great number of diseases have been recently modeled from specific mutations, such as immunodeficiency centromeric region instability and facial anomalies syndrome [64] and pancreatic cancer [65].

Likewise, it is worth mentioning that, in iPSCs from patients with mutated genes, CRISPR/Cas9 editing allows the knock-in of corrected alleles. Based on this background, several groups have proposed, in addition to modeling diseases, taking iPSCs to a new paradigm in cell therapy: the correction of genetic material* in vitro* before transplantation into patients, thus restoring lost functions in specific tissues. The main candidates for* in vitro* gene correction are monogenic diseases, such as *β*-thalassemia and hemophilia A. *β*-Thalassemia is a genetic disorder that is caused by mutations in the human hemoglobin beta (*HBB*) gene. CRISPR/Cas9 technology efficiently corrected the* HBB* mutations in patient-derived iPSCs, and when these cells differentiated into erythroblasts using a monolayer culture, gene-corrected iPSCs restored the expression of* HBB* and reduced the reactive oxygen production compared to the parental iPSCs line [66, 67]. Hemophilia A is an X-linked genetic disorder that is caused by mutations in the* F8* gene, which encodes blood coagulation factor VIII. Almost half of all severe hemophilia A cases result from chromosomal inversions. Interestingly, CRISPR/Cas9 nucleases were used to revert these chromosomal segments back to wild type in a mouse iPSC line that expressed the* F8* gene and functionally rescued factor VIII deficiency [68]. More importantly, gene editing excluded the modifications of potential off-targets (nonspecific sequences), which is an advantage compared to gene therapy based on viral vectors, which integrate randomly into multiple sites.

**Figure 1:**
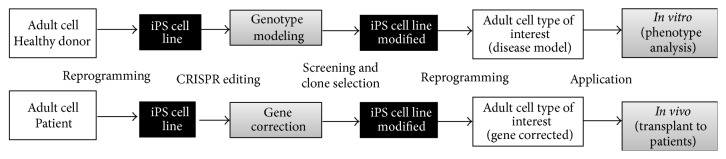
Methodology of gene editing for modeling or cell therapy based on iPSC and CRISPR/Cas9 technology.

While this strategy exhibits the same difficulties as standard reprogramming in translational medicine, an important advantage arises due to the restoration of lost functions in patients, making it an attractive scenario for the development of new protocols that guarantee higher levels of biosafety.  Figure 1 shows a general scheme of the methodology of iPSC and CRISPR/Cas9 technology that are used in gene editing for modeling or cell therapy.

## 8. Concluding Remarks

The mechanisms of action that are involved in cell reprogramming to pluripotency have begun to be elucidated, and future research will definitively focus on them. Although iPSCs resemble ESCs (e.g., they share the proliferation rate, self-renew, express the same molecular markers, and can differentiate into several cell types), the patterns of global gene expression and methylation differ deeply. Such differences currently concern the scientific community. However, iPSCs have already reached the clinical research phase thanks to exponential advances in the understanding of methylomes, proteomics, analysis of single cells, and so forth, technologies that previously seemed distant but have become reality.

Despite the main objective of iPSCs being clinical application, technologies based on miniorganoids are gaining interest. The possibility of obtaining* in vitro* organ-like structures drives a plethora of “clinical trials in Petri dishes” and promises the production of any desired organ in the lab for later transplantation. In addition, the association of iPSCs with CRISPR/Cas9 may lead to a unique combination of gene and cell therapies.

As shown in this minireview, the study of iPSCs not only began with great expectations, but is also accomplishing them every day.

## Figures and Tables

**Table 1:** Miniorganoids that have been obtained from iPSCs and associated disease modeling.

Miniorganoids	Disease modeled	References
Stomach	*Helicobacter pylori* infection	[25]
Gut	*Salmonella enterica* infection	[26, 27]
Liver	Lethal liver failure	[28]
Lungs	Cystic fibrosis	[29]
Brain	Alzheimer disease and Rhett Syndrome	[30, 31]
Heart	Cardiac failure	[32]
Eyes	Age-related macular degeneration	[33, 34]

**Table 2:** Obtaining and differentiating human iPSCs.

Precursor cells	Method of pluripotent stem cell induction	Type of cell-like or tissue-like produced	Reference
Human fibroblasts	Retrovirus	Neural and cardiac	[3]
Human fibroblasts	Retrovirus	Pancreatic islet	[34]
Human fibroblasts	Plasmid	Hepatic and cardiac	[44]
Commercial human cells	Without data	Retina	[45]
Thalassemia patient fibroblasts	Retrovirus	Hematopoietic	[46]
Human fibroblast	mRNA	iPSCs	[47]

**Table 3:** Therapeutic use of iPSCs in mice.

Precursor cells	Method of transfection	Type of cell or tissue produced	Results	Reference
Diabetic murine fibroblasts	Retrovirus	*β*-pancreatic	Reversion of hyperglycemia	[35]
Hemophilic murine fibroblasts	Retrovirus	iPSCs	Phenotypic reversion of hemophilia A	[36]
Embryonic fibroblasts	Lentivirus	Neurons	Partial reversion of damaged spinal chord	[37]
Humans Parkinson fibroblasts	Lentivirus	Clusters of neurons	Reduction of Parkinson disease in rats	[38]
Anemic mice fibroblasts	Retrovirus	Hematopoietic progenitors	Reversion of anemia	[39]
Human fibroblasts	Retrovirus	Pancreatic beta cells	Reversion of hyperglycemia	[40]
